# Pesticide Residue Behavior and Risk Assessment in Celery after Se Nanoparticles Application

**DOI:** 10.3390/foods10091987

**Published:** 2021-08-25

**Authors:** Lu Kang, Hejiang Liu, Duoyong Zhao, Canping Pan, Cheng Wang

**Affiliations:** 1 Innovation Center of Pesticide Research, Department of Applied Chemistry, College of Science, China Agricultural University, Beijing 100193, China; 96208zx@163.com; 2 Key Laboratory of Agro-Products Quality and Safety of Xinjiang, Institute of Agricultural Quality Standards and Testing Technology, Xinjiang Academy of Agricultural Sciences, Urumqi 830091, China; liuhejiang2025@163.com (H.L.); luckydyz@163.com (D.Z.)

**Keywords:** residue behavior, dissipation, celery, risk assessment, pesticides

## Abstract

This study investigates pesticide levels in celery, and compares their degradation, dissipation, distribution, and dietary risk after spraying with selenium (Se) nanoparticles. Abamectin, imidacloprid, acetamiprid, thiamethoxam, and lambda-cyhalothrin were sprayed at 1.6, 6.8, 2.0, 1.0, and 0.7 g a.i. ha^−1^ followed by a 2 g·ha^−1^ Se nanoparticle application during the growing period. Thiamethoxam, abamectin, imidacloprid, lambda-cyhalothrin, and acetamiprid in celery degraded following a first order kinetic model after 2 g·ha^−1^ Se nanoparticles application. With the exception of acetamiprid, the half-lives of thiamethoxam, abamectin, imidacloprid, and lambda-cyhalothrin were reduced from 2.4, 0.5, 1.2, 4.2 days without Se nanoparticles application to 1.4, 0.2, 0.9, 3.7 days with the addition of Se nanoparticles (2 g·ha^−1^), respectively. The chronic dietary exposure risk probability (RQc) and the acute dietary exposure risk probability (RQa) of celery after Se nanoparticles application were within acceptable limits for consumption except for abamectin.

## 1. Introduction

Celery (*Apium graveolens* L.) is a plant in the apiaceae family, which is rich in phenolic and antioxidant compounds [1,2]. The characteristic odor and flavor of celery consists mainly of a number of volatile compounds (terpenes, phthalates, and aldehydes) [3]. Flavonoids (apigenin, chrysin, luteolin) are subgroups of flavonoids that have been shown to have antioxidant, anti-inflammatory, anti-allergic, neuroprotective, and cardioprotective effects [4]. This biennial herb, originating from Europe, is now cultivated and consumed all over the world. The stalks (including leaves and petioles) are the main edible part of celery [5]. In China, the cultivation area of celery is around 550,000 hectares, ranking highest in the world [6].

Pesticides are widely used to control pests and diseases in soils and field crops [7,8] such as celery, although more recently, wider public concern has been aroused by their human health harm and environmental risk. Neonicotinoid pesticides are now the most widely used insecticides in China, and their continued global use can lead to accumulation in soil, posing potential ecological risks given their long half-life [9]. Recently, nanoparticles have been shown to effectively reduce and mitigate residues in crops by accelerating their degradation [10].

Nanomaterials are very small particles with surface properties that may bond and transport toxic chemical contaminants [11]. Se nanoparticles are synthesized utilizing various chemical, physical, and biological methods [12] and are less toxic and more biocompatible than selenates and selenites [13]. Se nanoparticles have a beneficial impact by increasing the photosynthetic ability of plant pigments and antioxidative metabolism, while reducing reactive oxygen species (ROS) formation. They have been shown to increase coffee yield and improve the nutritional quality of grains [14]. Se nanoparticles have also been shown to increase the expression of phytohormone synthesis genes, and enhance jasmonic, abscisic, and salicylic acid (SA) levels [15]. These plant hormones promote the degradation of toxic pesticide residues, such imidacloprid in cucumber by regulating the redox balance in the plant and promoting its metabolism by enhancing glutathione dependent detoxification [16]. Exogenous SA at 10 mg·L^−1^ was found to significantly reduce the half-lives of thiamethoxam, hymexazol and chlorantraniliprole, and inhibit accumulation of these pesticides in roots and leaves [17]. SA has also been shown to effectively slow down the accumulation of clothianidin, dinotefuran, and difenoconazole in roots and increase pesticide transport ability from root to leaf [18]. Accordingly, this study investigates whether Se nanoparticles can degrade pesticide residues in celery by inducing similar endogenous hormone changes.

Celery hosts several leaf-dwelling insect species, including *Philophylla heraclei L.* (Diptera: Tephritidae), *Spodoptera exigua Hübner* (Lepidoptera: Noctuidae), *Liriomyza trifolii Burgess* (Diptera: Agromyzidae), *Trialeurodes vaporariorum* (Westwood) (Hemiptera: Aleyrodidae), and *Listroderes costirostris Schönherr* (Coleoptera: Curculionidae) [19]. *Bemisia tabaci* have evolved resistance to many kinds of insecticides, including new neonicotinoids and conventional insecticides [20]. According to each pesticide manufacturer’s application guidelines, imidacloprid, abamectin, acetamiprid, thiamethoxam, and imidacloprid are all safe to apply during celery production. 

Of all neonicotinoids commonly measured in soil [21], acetamiprid, thiamethoxam, and imidacloprid were the most commonly detected neonicotinoid pesticides (found in 22 to 34% of samples) [22]. Abamectin and imidacloprid are touch-kill and stomach poisons, with low toxicity, fast photolytic breakdown and can control spider mite [23], aphid and leafhopper [24] pests. Thiamethoxam, a second-generation neonicotinoid insecticide, is also applied worldwide to control a variety of insects [25]. Lambda-cyhalothrin is a pyrethroid insecticide that acts on the nervous system of insects and regulates neural functions by interfering with sodium channels [26]. Clothianidin acts on insect acetylcholine receptors and affects insect central nervous systems [27,28].

The residue behavior of abamectin, chloranterbenamide, imidacloprid, and acetamiprid in the apple [29,30], grass carp [31], leek [32], tea [33], and cowpea [34] have been studied and reported in China and abroad. However, no systematic studies have been conducted to assess the residue distribution, degradation behavior, and associated dietary risk of clothianidam, imidacloprid, abamectin and chloranterbenamide in celery at the recommended dose and 1.5-fold the recommended dose. Furthermore, the effect of spraying 2 g·ha^−1^ Se nanoparticles as a mitigation measure to potentially reduce the residue distribution and improve the degradation behavior and dietary risk assessment of thiamethoxam, imidacloprid, avermectin, acetamiprid, and lambda-cyhalothrin in celery is also unreported. Moreover, clothianidin and chlorantraniliprole are as of yet unregulated pesticides, therefore, it is important to assess their safe use in celery.

This study aims to: (1) investigate the degradation behavior and residue distribution of imidacloprid, abamectin, thiamethoxam, lambda-cyhalothrin and acetamiprid in celery after spraying 2 g·ha^−1^ Se nanoparticles; (2) investigate the degradation behavior and residue distribution of clothianidin, imidacloprid, abamectin, chlorantraniliprole in celery and soil at the recommended dose and 1.5-fold the recommended dose; and (3) evaluate the dietary exposure of the above pesticides to provide guidelines for the safe application of (as yet unregulated) clothianidin and chlorantraniliprole in celery.

## 2. Materials and Methods

### 2.1. Chemicals and Reagents

Chromatography-grade methanol, acetonitrile, and formic acid were obtained from Fisher Scientific (Beijing, China). Analytical grade sodium chloride (NaCl) and anhydrous magnesium sulfate (MgSO_4_) were purchased from Sinopharm Chemical Reagent (Beijing, China). Analytical standard clothianidin, imidacloprid, abamectin, chlorantraniliprole, thiamethoxam, lambda-cyhalothrin, acetamiprid (purity 99.0%) were purchased from the Environmental Quality Supervision and Testing Center of the Ministry of Agriculture (Tianjin, China). Field trial pesticides were obtained from the following sources; Abamectin Emulsifiable Concentrates (EC, 3.2%) and imidacloprid Soluble Concentrate (SL, 20%) were supplied by Jingbo Agrochemical Technology Co. Ltd., Shandong, China; Abamectin Granule (GR, 0.5%) was supplied by Shenzhen Nuopu Agrochemical Co. Ltd., Shenzhen, China; Imidacloprid GR (5%) was supplied by Jiangsu Kesheng Group Co. Ltd., Jiangsu, China; Acetamiprid Water dispersible granules (WDG, 70%) was supplied by Shanghai Hulian Biological Pharmaceutical Co. Ltd., Shanghai, China; Thiamethoxam (12.6%) and lambda-cyhalothrin Suspension concentrate (SC, 9.4%) were supplied by Henan Xifunong Biological Technology Co. Ltd., Henan, China; Clothianidin (GR, 0.5%) was supplied by Nongdefeng Co. Ltd., Xinhui District, Jiangmen City, Guangdong Province, China; Chlorantraniliprole (SC, 5%) was supplied by FMC Corporation, Mainland, China; Se nanoparticles were supplied by Guilin Jiqi Group Co. Ltd., Guilin, China.

### 2.2. Field Trials and Sample Collection

Celery (Ventura variety, supplied by Beijing Genqing Seed Co., Ltd., Beijing, China) was grown in the Xinjiang test facility greenhouse in 2020 with a temperature at around 10 °C. The soil type was ash desert soil, with a pH of 7.9, and organic matter content was 3.6%. The field experiments were designed to investigate the residue persistence of clothianidin, imidacloprid, abamectin and chlorantraniliprole in celery under greenhouse conditions in China. The field trials were designed in accordance with the Guideline on Pesticide Residue Trials [35]. Two trials were made to compare the degradation safety of pesticide residues in celery after applying different pesticide doses. 

For the first trial, clothianidin, imidacloprid, abamectin, and chlorantraniliprole granules were applied onto the soil at the celery roots at the recommended dose and 1.5-fold the recommended dose. Pesticide labeling information and recommended dose was shown in Table S1. Representative samples of the celery were collected randomly at 14, 21, 28, and 50 days after pesticide application to analyze for clothianidin. Representative samples of the celery were collected randomly at 14, 21, 28, 60, and 77 days after pesticide application to analyze for imidacloprid and abamectin. Chlorantraniliprole was applied 30 days before celery was harvested. Representative samples of celery were collected randomly at 2 h and 1, 3, 5, 7 and 14 days after spraying with chlorantraniliprole.

For the second trial, representative samples of celery were collected randomly at 2 h and 1, 3, 5, 7 and 14 days after the spraying with thiamethoxam, abamectin, imidacloprid, lambda-cyhalothrin and acetamiprid. Table S2 shows pesticide labeling information and recommended dose combined with 2 g·ha^−1^ Se nanoparticles. After pesticide spray application, the celery also received a spray application of 2 g·ha^−1^ Se nanoparticles for each experimental plot, while control plots (with no Se nanoparticles application) were also included. The area of each plot was 20 m^2^, with three replicates. A buffer zone was utilized between the different treatment varieties to avoid cross-contamination. At least 1 kg of celery was harvested from each plot, homogenized and maintained at −20 °C before further analysis. 

### 2.3. Method Validation

The performance of the proposed analytical method was verified on celery using the following parameters: linearity, limit of quantification (LOQs), accuracy, and precision. This method validation study was undertaken by comparing matrix-matched standard curves with celery matrix extracts. The LOQ is the lowest spiked level of the validation meeting these method performance acceptability criteria. The linearity of pesticide residue analysis method should be above 0.99, the recovery rate of pesticide should be between 70 and 120%, and the RSD should be between 0 and 20%. The accuracy and precision of the analytical method was measured from recovery tests with 3 spiked levels of pesticides (0.01, 0.05 and 0.1 mg kg^−1^) in 5 replicates. The concentration of pesticide residue was achieved with acceptable accuracy by the application of a complete analytical method in accordance with the European criteria guidelines established by SANTE/12682/2019 [36]. 

### 2.4. Extraction and Purification

Up to three kilograms of celery was collected in an S-shaped pattern across each plot, then homogenized to a pulp with a blender and stored at −18 °C until analysis. Soil samples were also collected, sieved to 1 mm and stored at −18 °C. Fifteen grams of each celery sample were weighed into a 50 mL plastic centrifuge tube, and 30 mL acetonitrile and 5–7 g sodium chloride were added [29]. The tube contents were homogenized for 1 min using a high-speed tissue homogenizer at 16,099× *g* and then centrifuged for 1 min using a high-speed centrifuge at 11,180× *g* until the acetonitrile phase and the aqueous phase separated. The pretreatment extraction method for pesticide residues in soil samples was the same as that of the celery extraction. According to the different analytical instruments or detectors required to analyze each pesticide, further processing was as follows:

(1) 0.5 mL of the upper acetonitrile extract was added into a solution of 0.5 mL methanol and water (1:1). The mixture was eddy mixed and filtered through a 0.22 μm organic microporous filtration membrane for LC-MS/MS.

(2) 10 mL of the supernatant was reduced to near dryness for purification in a 150 mL round bottom flask in a water bath at 40 °C. Around 30 mL of acetonitrile and toluene (3:1) mix is used as an eluent solution for solid phase extraction. First, 5 mL of the eluent solution was used to pre-wash the graphite amino series-to-solid phase purification column. When the level of eluent solution reached the small column filter, about 1.5 mL of eluent solution was used to collect the residue from the round bottom flask bottle and then quickly transferred to the purification column. This step was repeated 3 times (each time, the new eluent was only transferred when the level of eluent solution reached the small column filter). The remaining eluent was poured into the round bottomed flask and gradually transferred to the purification column. After purification, the effluent (collected in another 150 mL round bottomed flask) was evaporated to near dryness in a 40 °C water bath. 2 mL of *n*-hexane was added, and the solution was passed through a 0.22 μm organic microporous filtration membrane before GC-MS/MS. Lambda-cyhalothrin was analyzed by GC-MS/MS, the remaining six pesticides were analyzed by UPLC-MS/MS.

### 2.5. LC-MS/MS Conditions

Pesticide extracts from celery and soil samples were analyzed using UPLC Xevo TQ-S micro (Waters, Milford, MA, USA). Chromatographic column: BEH C18 column 100 A (50 mm× 1.7 mm, particle size: 5 μm), Waters Corporation, USA; column temperature was 40 °C, and the liquid chromatography mobile phase and gradient elution conditions are shown in Table 1. Sampling volume: 10 uL; Ion source: ESI, scanning mode: positive and negative ion scanning; ion source temperature was 150 °C, desolventing temperature was 350 °C, N_2_ gas flow rate was: 650 L/h, N_2_ conical gas flow rate was 250 L/h, and the mobile phase flow rate was 0.20 mL/min. The mass spectrometry parameters for 6 target compounds, such as qualitative ion pair, quantitative ion pair, collision energy (Ce), and the conical voltage, are shown in Table S3.

### 2.6. GC-MS/MS Conditions

Celery samples were analyzed using 7000 B GC/MS Triple Quad (Agilent Technologies, Santa Clara, CA, USA) with autosampler. Sampling mode: no shunt injection; Inlet temperature: 250 °C; Injection volume: 1 μL; Chromatography was performed on a HP-5 column (30 m × 250 μm, 0.25 μm). Column heating procedure: 70 °C for 1 min, 20 °C/min to 270 °C, maintain: 8 min; Transmission line temperature: 280 °C; Carrier gas: helium, 1.5 mL/min; Quenching gas (helium): 2.25 mL/min, collision gas (nitrogen): 1.5 mL/min.

Ion source: electron bombardment ion source (EI), temperature 230 °C; Four-stage rod temperature: 150 °C; Emission current 50 μA; Masshunter workstation was used for instrument control and data processing. Scanning method: multiple reaction monitoring (MRM). The parameters of lambda-cyhalothrin mass spectrum are shown in Table S3.

### 2.7. Dissipation Kinetics of Pesticides

The pesticide dissipation dynamics of celery for the two treatments were evaluated in this study. Residue dissipation behaviors of the 7 pesticides were analyzed using a first order kinetic equation as follows [37,38]:

C_t_ = C_0_ e^−kt^ (1)

t_1/2_ = ln2/k (2)

In this equation, C_t_ is the residual concentration of pesticide at t (day), C_0_ (mg·kg^−^^1^) is the initial concentration of pesticide, and k is the dissipation rate constant. The t_1/2_ is pesticide half-life of pesticide dissipation.

### 2.8. Risk Assessment

To verify the limits for safe application of clothianidin, imidacloprid, abamectin, chlorantraniliprole, thiamethoxam, lambda-cyhalothrin, and acetamiprid, the health risk was quantified based on RQc [39] for chronic dietary exposure risk probability and RQa for acute dietary exposure risk probability, respectively.

(3) $$\mathrm{NEDI}=\sum \left(STMR_{i}\times \mathrm{Fi}\right)$$

(4) RQc%=NEDI/( ADI × bw )×100

(5) RQa%=NEDI/( ARfD × bw )×100

Here, NEDI (mg·kg^−^^1^ bw) is defined by the national estimated daily intake, STMR_i_ is the supervised trial median residue and F_i_ is the dietary reference intake of a specific food for the general population [40]. ADI (mg·kg^−^^1^ bw) was the acceptable daily intake and ARfD (mg·kg^−^^1^ bw) was acute reference dose. bw refers to the mean body weight of a person in China (63 kg).

## 3. Results and Discussion

### 3.1. Method Validation

According to methods approved by the Ministry of Agriculture and Rural Affairs of China (GB/T 20769-2008), clothianidine and imidacloprid residues in celery and soil, and thiamethoxam and acetamiprid residues in celery are only a few of the 450 pesticides and related chemical residues that are routinely analyzed in fruits and vegetables using LC-MS/MS. Abamectin residues [31] in celery and soil, and chlorantraniliprole residues [41] in celery were determined from literature methods. Lambda-cyhalothrin was analyzed in celery according to GC-MS/MS methods [42] (GB 23200.113-2018, Ministry of Agriculture and Rural Affairs of China) which can determine 208 pesticide residues and their metabolites in plant origin foods.

The analytical methods were verified by linearity, limit of quantification (LOQ), accuracy, and precision. Linearity was determined using a standard solution with a mix of five pesticide concentrations (0.01 to 5.0 mg·kg^−^^1^). Linearity calibration curves were constructed between the integrated chromatographic peak area and the corresponding concentration. All standard curves had good linear relationship, and the correlation coefficients (R^2^) were ≥0.99.

The field trial pesticides were added to the standard matrix to confirm the extraction efficiency [43,44]. In order to evaluate accuracy and precision, recovery experiments were performed. Blank samples were replicated 5 times with 3 spiked pesticide concentration levels (0.01 to 0.1 mg·kg^−^^1^). Before extraction, 3 concentrations of clothianidam, imidacloprid, thiamethoxam, acetamiprid, chlorantraniliprole, and abamectin were added into a homogenized celery sample to obtain a spiked sample. A spiked celery sample with lambda-cyhalothrin was analyzed separately. The precision of the analysis method was measured by the relative standard deviation (RSD) [40]. The clothianidin, imidacloprid, thiamethoxam, and acetamiprid pesticide recovery levels from celery were 72.0 to 80.0%, 97.4 to 101.2%, 93.6 to 105.2%, and 80.2 to 90.3% at each of the three spiked concentration levels, respectively. The abamectin, chlorantraniliprole, and lambda-cyhalothrin pesticide recoveries from celery were 65.5 to 69.8%, 94.1 to 115.8% and 92.3 to 105.1%, respectively (Table 2). The clothianidin, imidacloprid and abamectin pesticide recoveries from soil were 79.2 to 83.1%, 85.2 to 106.2% and 68.1 to 70.2%, respectively (Table 3). The results showed that, except for the low recovery rate of abamectin, the above methods met the requirements of Guide to Pesticide Residue Tests in China [45] (NY/T 788-2018). 

The results showed that the limits of quantification for thiamethoxam, imidacloprid, clothiamidam, acetamiprid, lambda-cyhalothrin, abamectin, and chlorantraniliprole were 0.01 mg·kg^−^^1^. The analytical method showed good sensitivity, accuracy, and precision, and was found to be suitable for the determination of clothiamidam, imidacloprid, thiamethoxam, acetamiprid, chloranterianamide, abamectin, and lambda-cyhalothrin in celery and soil. 

### 3.2. Residue Levels in Celery

The limit of quantitation (LOQ) was assigned as 0.01 mg kg^−1^. A maximum residue limit (MRL) for clothianidin, imidacloprid, abamectin, and chlorantraniliprole in celery was set at 0.04, 6, 0.03, 7 mg kg^−1^ according to Codex regulations (http://www.fao.org/fao-who-codexalimentarius/codex-texts/dbs/pestres/pesticides/pt/ (accessed on 5 April 2021)) and 0.01, 0.5, 0.01, 0.06 mg kg^−1^ for EU regulations (https://ec.europa.eu/food/plant/pesticides/eu-pesticides-database/mrls/?event=search.pr (accessed on 5 April 2021)).Clothianidin and chlorantraniliprole are presently unregistered for use on celery and leafy vegetables, so a further objective of the study was to verify the safe use of these two pesticides for celery. Table 4 shows the initial and final residue levels of clothianidam, imidacloprid, abamectin, and chlorantraniliprole at the recommended dose and 1.5-fold the recommended dose. The initial residue content of clothianidin was 0.108 mg kg^−1^ at 1.5-fold the recommended dose of 23.6 g a.i. ha^−1^, which was higher than 0.06 mg kg^−1^ at the recommended dosage of 16.8 g a.i. ha^−1^. The final residue level of clothianidin was less than LOQ at 1.5-fold the recommended dose of 23.6 g a.i. ha^−1^, which was lower than 0.013 mg kg^−1^ at the recommended dose of 16.8 g a.i. ha^−1^. The initial residue level of imidacloprid was 0.029 mg kg^−1^ at 1.5-fold the recommended dose of 84.4 g a.i. ha^−1^, which was higher than 0.007 mg kg^−1^ at the recommended dose of 56.2 g a.i. ha^−1^. However, the final residue level of imidacloprid was 0.404 mg kg^−1^ at the recommended dose of 56.2 g a.i. ha^−1^, which was higher than 0.246 mg kg^−1^ at 1.5-fold the recommended dose of 84.4 g a.i. ha^−1^, the specific reason for this anomaly is yet unknown. The initial residue level of abamectin was 0.033 mg kg^−1^ at 1.5-fold the recommended dose of 19.7 g a.i. ha^−1^, which was higher than 0.021 mg kg^−1^ at the recommended dose of 16.9 g a.i. ha^−1^. The initial residue level of chlorantraniliprole was 4.958 mg kg^−1^ at 1.5-fold the recommended dose of 10.1 g a.i. ha^−1^, which was higher than 2.701 mg kg^−1^ at the recommended dose of 6.8 g a.i. ha^−1^. The final residue level of clothianidin was 1.847 mg kg^−1^ at 1.5-fold the recommended dose of 10.1 g a.i. ha^−1^, which was higher than 1.784 mg kg^−1^ at the recommended dose of 6.8 g a.i. ha^−1^. All celery with 1.5-fold the recommended dose of clothianidin, abamectin and chlorantraniliprole had higher residue levels than at the recommended dose that except for imidacloprid, for both the initial and final residues in celery.

Table 5 shows the initial and final residues of thiamethoxam, abamectin, imidacloprid, acetamiprid, and lambda-cyhalothrin applied to celery at the recommended dose (control treatment) and the same pesticides combined with an application of 2 g·ha^−1^ Se nanoparticles (Se nanoparticles treatment). After the application of Se nanoparticles, the initial residue levels of thiamethoxam, abamectin, imidacloprid and acetamiprid of celery collected after 2 h were found to be lower than the Chinese maximum residue limit (MRL), although lambda-cyhalothrin was higher than the Chinese maximum residue limit (MRL) in both treatments. The application of Se nanoparticles to celery significantly reduced the residue levels of imidacloprid and lambda-cyhalothrin after 14 days. Se nanoparticles had no significant effect on residue digestion of acetamiprid after 7 days. With the exception of the control treatment lambda-cyhalothrin, the final residue levels of the other pesticides in Se nanoparticles treated celery were below the maximum residue limits after 14 set by China, Codex Alimentarius Commission and the European Union. The recommended doses for thiamethoxam, abamectin, imidacloprid, lambda-cyhalothrin and acetamiprid were 1.0 g a.i.ha^−^^1^, 1.6 g a.i. ha^−^^1^, 6.8 g a.i. ha^−^^1^, 0.7 g a.i. ha^−^^1^, and 2.0 g a.i. ha^−^^1^, respectively. With the exception of acetamiprid, the original deposition amount (at 2 h) of thiamethoxam, abamectin, imidacloprid, and lambda-cyhalothrin in celery treated with Se nanoparticles were higher than the control treatment. The original deposition amount (at 2 h) of thiamethoxam, abamectin, imidacloprid and lambda-cyhalothrin in celery treated by Se nanoparticles were 0.131 mg kg^−1^, 0.351 mg kg^−1^, 4.613 mg kg^−1^ and 0.709 mg kg^−1^, respectively, which were higher than 0.101 mg kg^−1^, 0.219 mg kg^−1^, 4.446 mg kg^−1^, 0.687 mg kg^−1^ in the control treatment. The original deposition amount of acetamiprid in celery treated with Se nanoparticle was the same as in the control treatment. A possible reason is that the application of Se nanoparticles changed the celery’s surface texture and increased the adhesion of thiamethoxam, abamectin, imidacloprid, and lambda-cyhalothrin.

### 3.3. Dissipation Dynamics of Pesticide Residues in Celery

**Figure 1:**
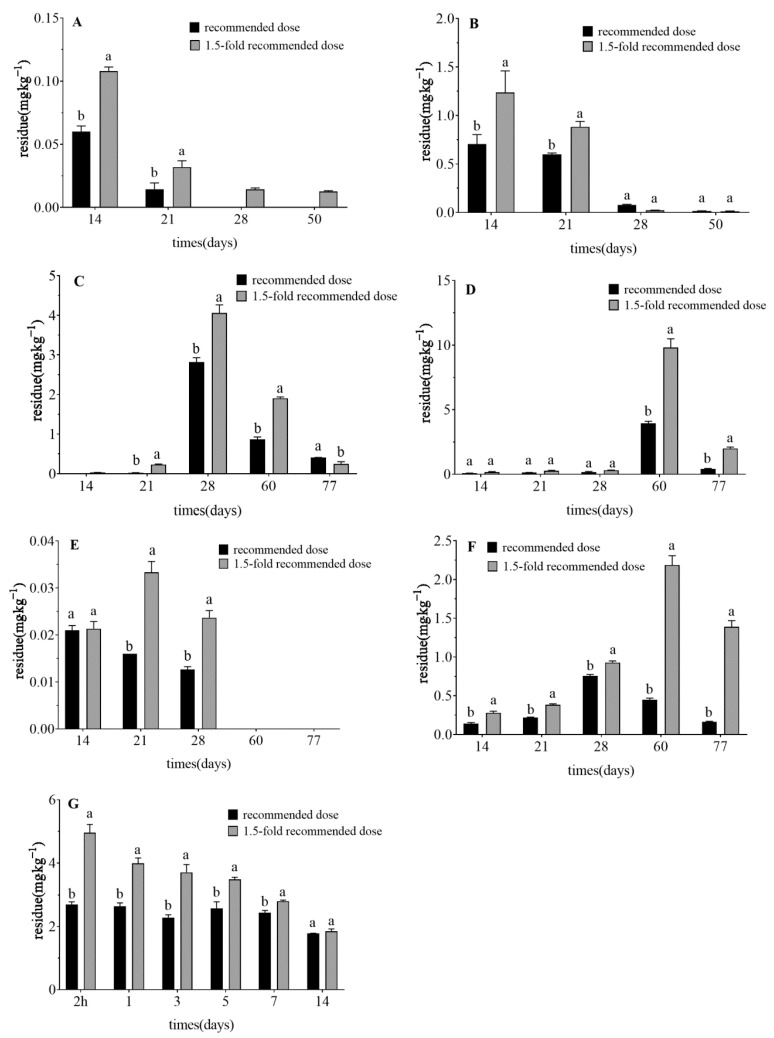
Dissipation rates of (**A**) clothianidin in celery, (**B**) clothianidin in soil, (**C**) imidacloprid in celery, (**D**) imidacloprid in soil, (**E**) abamectin in celery, (**F**) abamectin in soil, (**G**) chlorantraniliprole in celery after application of the pesticide at the recommended dose and 1.5-fold the recommended dose. Different letters across treatments indicate significant differences at *p* < 0.05.

Pesticide dissipation rates and their half-lives are important indices to evaluate pesticide behavior in the environment [46]. Table 6 and Figure 1 show the degradation curves, half-lives, and dissipation rates of clothianidin, imidacloprid, abamectin, and chlorantraniliprole at the recommended dose and 1.5-fold the recommended dose. Dissipation kinetics of the four pesticides in celery applied at the recommended dose were as follows: Ct = 0.059 × 10^−0.04t^ (clothianidin, R^2^ = 0.78, t_1/2_ =16.9 days); Ct = 8.619 × 10^−0.04t^ (imidacloprid, R^2^ = 0.99, t_1/2_ = 46.5 days); Ct = 0.035 × 10^−0.04t^ (abamectin, R^2^ = 0.99, t_1/2_ = 33.1 days); Ct = 2.766 × 10^−0.03t^ (chlorantraniliprole, R^2^ = 0.98). Dissipation kinetics of the same four pesticides in celery applied at 1.5-fold the recommended dose were as follows: Ct = 0.119 × 10^−0.05t^ (clothianidin, R^2^ = 0.81, t_1/2_ = 15.8 days); Ct = 22.670 × 10^−0.05t^ (imidacloprid, R^2^ = 0.91, t_1/2_ = 45.5 days); Ct= 0.041 × 10^−0.02t^ (abamectin, R^2^ = 0.97, t_1/2_ = 39.8 days); Ct = 4.614 × 10^−0.07t^ (chlorantraniliprole, R^2^ = 0.97). The degradation of clothianidin, imidacloprid, abamectin and chlorantranzoamide in celery at the recommended dose and 1.5-fold the recommended dose conformed with a first-order kinetics equation. The rate of abamectin dissipation was previously noted as 6 days on squash fruits [47]. In this study, the half-life of abamectin in celery was longer, which was related to the fact that abamectin granules, rather than a dispersive spray solution, were used in this experiment.

The half-life of clothianidin in celery and soil at the recommended dose was found to be greater than 1.5-fold the recommended dose (Table 6). The half-life of imidacloprid in celery at the recommended dose was also higher than 1.5-fold the recommended dose, while the half-life of imidacloprid in soil was lower than 1.5-fold the recommended dose. Dissipation kinetics of these three pesticides in soil at the recommended dose were as follows: Ct = 3.632 × 10^−0.11t^ (clothianidin, R^2^ = 0.96, t_1/2_ = 21.2days); Ct = 23.988 × 10^−0.07t^ (imidacloprid, R^2^ = 0.99, t_1/2_ = 37.9 days); Ct = 1.907 × 10^−0.03t^ (abamectin, R^2^ = 0.94, t_1/2_ = 55.9 days). Dissipation kinetics of the three pesticides in soil at 1.5-fold the recommended dose were as follows: Ct = 5.636 × 10^−0.13t^ (clothianidin, R^2^ = 0.86, t_1/2_ = 17 days); Ct = 75.784 × 10^−0.07t^ (imidacloprid, R^2^ = 0.98, t_1/2_ = 40.3 days); Ct = 3.611 × 10^−0.02t^ (abamectin, R^2^ = 0.99, t_1/2_ = 70.3 days). The degradation of clothianidin, imidacloprid and abamectin in soil at the recommended dose and 1.5-fold the recommended dose conformed with a first-order kinetics equation. The half-life of abamectin in celery and soil at the recommended dose was lower than 1.5-fold the recommended dose. Chlorantraniliprole was sprayed at either 2- or 3-fold the recommended dose on the pesticide label respectively, and the dissipation of chlorantraniliprole residue was monitored for 3 days until the celery was harvested. The half-life of chlorantraniliprole (calculated according to the residue dissipation) was not representative, so is not discussed further.

### 3.4. Dissipation Dynamics of Five Pesticide Residues in Celery by Se Nanoparticles

**Figure 2:**
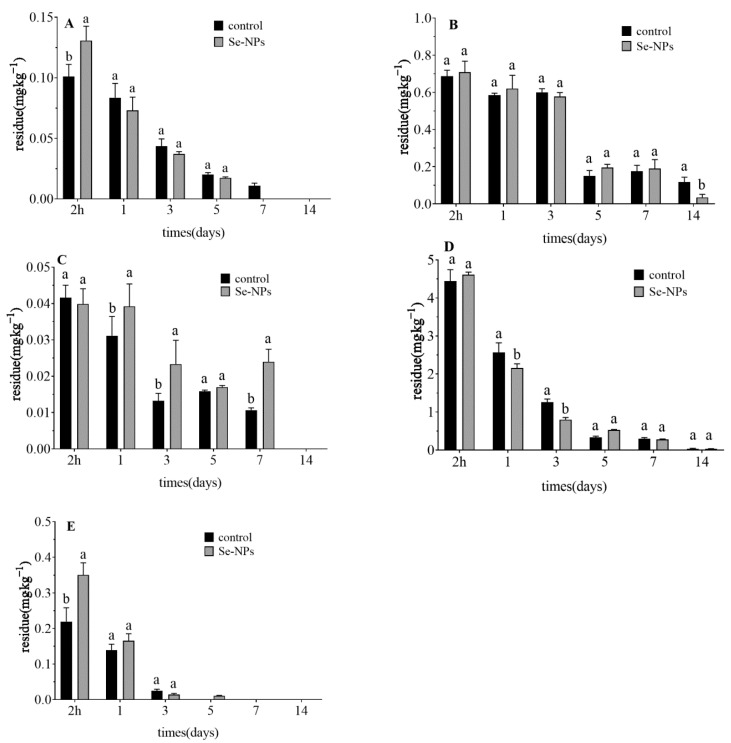
Dissipation rates of (**A**) thiamethoxam, (**B**) lambda-cyhalothrin, (**C**) acetamiprid, (**D**) imidacloprid and (**E**) abamectin on celery after application of pesticides at the recommended dose combined with 2 g·ha^−1^ Se nanoparticles. Different letters across treatments indicate significant differences at *p* < 0.05.

In order to investigate the effect of Se nanoparticles on the residue behavior of five pesticides on celery, pesticide concentrations of celery at different growth stages were measured at 2 h and 1, 3, 5, 7, 10, and 14 days after Se nanoparticles application. Table 7 and Figure 2 show the degradation curves, half-lives, and dissipation rates of thiamethoxam, abamectin, imidacloprid, acetamiprid, and lambda-cyhalothrin at the recommended dose combined with a spray application of 2 g·ha^−1^ Se nanoparticles. The original deposition amounts of these 5 active ingredients in 4 pesticide preparations were obtained respectively. Table 7 describes the residue dissipation behavior on the 5 pesticides used in the trial after treatment with Se nanoparticles.

The dissipation kinetics of five pesticides in celery at the recommended dose were as follows: Ct = 0.109 × 10^−0.32t^ (thiamethoxam, R^2^ = 0.99, t_1/2_ = 2.4 days); Ct = 0.093e × 10^−0.34t^ (abamectin, R^2^ = 0.86, t_1/2_ = 0.5 days); Ct = 3.365 × 10^−0.34t^ (imidacloprid, R^2^ = 0.98, t_1/2_ = 1.2 days); Ct = 0.036 × 10^−0.19t^ (acetamiprid, R^2^ = 0.97, t_1/2_ = 2.9 days); Ct = 0.595 × 10^−0.13t^ (lambda-cyhalothrin, R^2^ = 0.86, t_1/2_ =4.2 days). Two days after spraying 2 g·ha^−1^ Se nanoparticles, the dissipation kinetics of the five pesticides in celery were as follows: Ct = 0.103 × 10^−0.33t^ (thiamethoxam, R^2^ = 0.99, t_1/2_ = 1.4 days); Ct = 0.198 × 10^−0.52t^ (abamectin, R^2^ = 0.98, t_1/2_ = 0.2 days); Ct = 0.042 × 10^−0.14t^ (imidacloprid, R^2^ = 0.95, t_1/2_ = 5.3days); Ct = 0.109 × 10^−0.32t^ (acetamiprid, R^2^ = 0.99, t_1/2_ = 2.4 days); Ct = 0.797 × 10^−0.22t^ (lambda-cyhalothrin, R^2^ = 0.98, t_1/2_ = 3.7 days). The degradation of thiamethoxam, imidacloprid, abamectin, lambda-cyhalothrin and chlorantraniliprole in celery after treatment with the recommended dose and spraying 2 g·ha^−1^ Se nanoparticles conformed to a first-order kinetics equation. After 14 days, the final residue level of lambda-cyhalothrin was higher than the EU maximum residue limit (MRL), but the residue level of lambda-cyhalothrin treated with Se nanoparticles was less than the control treatment, and the half-life of lambda-cyhalothrin was 0.5 days shorter than the control treatment. 

Apart from acetamiprid, the application of Se nanoparticles to celery shortened the residue half-lives of thiamethoxam, abamectin, imidacloprid and lambda-cyhalothrin. Under greenhouse conditions, the residue levels of celery samples at both recommended dose and 1.5-fold the recommended dose after 7 days were lower than the Chinese MRL. According to residue monitoring throughout the field experiment, safe harvest of celery was possible only 22 days and 31 days after the application of clothianidin granules and chlorantraniliprole spray at 1.5-fold the recommended dose, respectively. These more persistent individual residue behaviors may arise from different external conditions such as crop species or climate conditions (temperature, viscosity) at spraying time [40]. Pre-harvest withholding intervals are usually set at seven days to ensure that pesticide residues are below the MRL [48]. However, half-lives of lambda-cyhalothrin and thiamethoxam have previously been noted from 7.01 d to 17.3 d [30], the half-life of abamectin was found to be 1.02 days in strawberry [49]. The dissipation rates of acetamiprid and lambda-cyhalothrin have previously been shown to be a first-order rate kinetic equation, with the half-lives of these chemicals in pomegranate fruits from 9.2 to 13 days and 13.5 to 17 days in the leaves [50]. 

### 3.5. Dietary Intake Risk Assessment for Celery

#### 3.5.1. Chronic Dietary Exposure Risk Assessment for Celery

NEDIs and RQs were calculated according to Chinese dietary patterns. The chronic dietary risk assessment of clothianidam, imidacloprid, abamectin and chlorantraniliprole in celery at recommended dose and 1.5-fold the recommended dose is shown in Table 8. According to the principle of maximum dietary risk, chronic dietary risk assessment was conducted for MRLs of celery. The ADI and ARfD were obtained from the MRLs of Pesticides in Food [51] (GB 2763-2019) and the JMPR report, respectively. When calculating the NEDI value corresponding to each food group, the MRLs referred to Chinese standards. The STMR of imidacloprid, abamectin, clothianidin, and chlorantraniliprole in celery were used as the reference MRL’s for national chronic dietary risk assessment where celery belongs to the dark vegetable group. STMR was used to calculate the NEDI of clothianidin, imidacloprid, abamectin and chlorantraniliprole at the recommended dose and 1.5-fold the recommended dose. Abamectin and its isomeric 8, 9-Z avermectin B_1_a, a photolytic product of avermectin B_1_a, were defined as chemical residues in dietary risk assessment [52].

As illustrated in Table 8, the dietary risk of celery was acceptable for consumers apart from celery treated with abamectin granules. The application of clothianidam, imidacloprid, and chlorantraniliprole at the recommended dose and 1.5-fold the recommended does not raise a dietary risk to ordinary Chinese consumers. The chronic dietary risk assessment of thiamethoxam, acetamiprid, imidacloprid, abamectin, and lambda-cyhalothrin in celery after the recommended dose and spraying with 2 g·ha^−1^ Se nanoparticles is shown in Table 9. STMR was used to calculate the recommended dose and NEDI of thiamethoxam, acetamiprid, imidacloprid, abamectin, and lambda-cyhalothrin after spraying with 2 g·ha^−1^ Se nanoparticles. As illustrated in Table 9, the dietary risk for celery was acceptable for consumers, apart from abamectin emulsion sprays. Abamectin is considered to be a dangerous pesticide with a variety of cytotoxic and genotoxic effects on non-target organisms [53]. The rate of dissipation in abamectin was previously noted to be 6 days after application on squash fruits [47].

#### 3.5.2. Acute Dietary Exposure Risk Assessment for Celery

This study also assessed the risk of acute dietary exposure to imidacloprid, abamectin, acetamiprid, thiamethoxam, and lambda-cyhalothrin in celery. According to the JMPR report, the ARfD values of imidacloprid, acetamiprid, clothianidin, thiamethoxam, and lambda-cyhalothrin were 0.4 mg·kg^−^^1^ bw, 0.1 mg·kg^−^^1^ bw, 0.6 mg·kg^−^^1^ bw, 1.0 mg·kg^−^^1^ bw, 0.02 mg·kg^−^^1^ bw, respectively. ARfD of abamectin and chlorantraniliprole was not calculated for these two pesticides. The highest residues (HR) for clothianidin and imidacloprid in celery for these experimental trials were 0.014 mg·kg^−^^1^, 0.032 mg·kg^−^^1^, 2.81 mg·kg^−^^1^, and 4.06 mg·kg^−^^1^ at the recommended dose and 1.5-fold the recommended dose, respectively. The RQa of clothianidin, imidacloprid, and chlorantraniliprole in celery were all less than 100%. The HR of thiamethoxam, acetamiprid, imidacloprid, and lambda-cyhalothrin were 0.101 mg·kg^−^^1^, 0.131 mg·kg^−^^1^, 0.042 mg·kg^−^^1^, 0.040 mg·kg^−^^1^, 4.446 mg·kg^−^^1^, 4.613 mg·kg^−^^1^, 0.687 mg·kg^−^^1^, and 0.709 mg·kg^−^^1^ after spraying with 2 g·ha^−1^ Se nanoparticles, respectively. The RQa of thiamethoxam, acetamiprid, imidacloprid, and lambda-cyhalothrin in celery were all less than 100%, respectively. These results suggest there is a negligible risk of acute dietary exposure to clothiamethoxam, imidacloprid, thiamethoxam, acetamiprid, and lambda-cyhalothrin at these application rates.

## 4. Conclusions

GC-MS/MS and UPLC-MS/MS were used to measure the recovery, precision, accuracy and degradation behavior of lambda-cyhalothrin, clothianidin, imidacloprid, abamectin, chlorantraniliprole, thiamethoxam, and acetamiprid residues in celery. Results showed that, apart from acetamiprid, the half-lives of thiamethoxam, abamectin, imidacloprid and lambda-cyhalothrin were 2.4, 0.5, 1.2, 4.2 days and 1.4, 0.2, 0.9, and 3.7 days for the control and a further Se nanoparticles treatment (2 g·ha^−1^), respectively. According to STMR, chronic and acute dietary exposure risks were assessed for clothianidin, imidacloprid, abamectin, chlorantraniliprole, thiamethoxam, lambda-cyhalothrin, and acetamiprid in celery. The dietary risk of celery was acceptable for consumers apart from abamectin, whether emulsion sprays or granules were used. This study provides a valuable reference for the safe and reasonable application of recommended doses of imidacloprid, abamectin, thiamethoxam, and lambda-cyhalothrin for celery cultivation in China.

## Figures and Tables

**Table 1:** Mobile phase and gradient elution conditions.

Time (min)	Flow Velocity (mL/min)	Phase A: Methanol (%)	Phase B: Water (5 mmol/L Ammonium Acetate + 0.1% Formic Acid) (%)
0.0	0.2	5	95
3.0	0.2	5	95
7.0	0.2	95	5
16.0	0.2	95	5
18.0	0.2	5	95
20.0	0.2	5	95

**Table 2:** Method validation of pesticides in a celery matrix.

Pesticide	Linear Range (mg·kg^−1^)	Correlation Coefficient (R^2^)	LOQ (mg·kg^−1^)	Recovery (%) ± RSDs (%), Spiked Level (mg·kg^−1^)		
				0.01	0.05	0.1
Clothianidin	0.01–5.0	0.9943	0.01	72.0 ± 1.7	80.0 ± 2.2	74.1 ± 3.0
Imidacloprid	0.01–5.0	0.9982	0.01	101.0 ± 4.8	101.2 ± 3.8	97.4 ± 3.6
Abamectin	0.01–5.0	0.9975	0.01	66.3 ± 3.3	65.5 ± 2.2	69.8 ± 2.4
Chlorantraniliprole	0.01–5.0	0.9983	0.01	94.1 ± 3.0	115.8 ± 3.5	112.7 ± 2.8
Thiamethoxam	0.01–5.0	0.9921	0.01	105.2 ± 3.2	97.1 ± 4.0	93.6 ± 4.5
Lamda-cyhalothrin	0.01–5.0	0.9954	0.01	102.4 ± 6.2	92.3 ± 8.2	105.1 ± 5.6
Acetamiprid	0.01–5.0	0.9961	0.01	80.2 ± 9.2	88.6 ± 10.1	90.3 ± 8.5

**Table 3:** Method validation of pesticides in a soil matrix.

Pesticide	Linear Range (mg·kg^−1^)	Correlation Coefficient (R^2^)	LOQ (mg·kg^−1^)	Recovery (%) ± RSDs (%), Spiked Level (mg·kg^−1^)		
				0.01	0.05	0.1
Clothianidin	0.01–10.0	0.9965	0.01	79.2 ± 3.4	83.1 ± 4.6	82.0 ± 5.2
Imidacloprid	0.01–10.0	0.9959	0.01	85.2 ± 6.2	91.6 ± 2.9	106.2 ± 4.3
Abamectin	0.01–10.0	0.9982	0.01	68.1 ± 2.9	69.7 ± 3.0	70.2 ± 3.8

**Table 4:** Residue levels at different pre-harvest intervals after application of the recommended dose and 1.5-fold the recommended dose.

Dose	Pesticide	PHI (Days) (mg·kg^−1^)						MRL (mg·kg^−1^)			Recommended PHI (Days)
		14	21	28	50	60	77	China	CAC	EU	
Recommended dose	Clothianidin (GR)	0.06 ± 0.00 ^b^	0.014 ± 0.00 ^b^	<LOQ	<LOQ	-	-	0.04	0.04	0.01	10
1.5-fold recommended dose		0.11 ± 0.00 ^a^	0.032 ± 0.01 ^a^	0.014 ± 0.00	0.013 ± 0.00	-	-				22
Recommended dose	Imidacloprid (GR)	<LOQ	0.024 ± 0.00 ^b^	2.81 ± 0.12 ^b^	-	0.86 ± 0.06 ^b^	0.4 ± 0.01 ^a^	5	6	0.5	14
1.5-fold recommended dose		0.029 ± 0.00	0.23 ± 0.01 ^a^	4.06 ± 0.21 ^a^	-	1.9 ± 0.03 ^a^	0.25 ± 0.06 ^b^				31
Recommended dose	Abamectin (GR)	0.021 ± 0.00 ^a^	0.016 ± 0.00 ^b^	0.013 ± 0.00 ^b^	-	<LOQ	<LOQ	0.05	0.03	0.01	9
1.5-fold recommended dose		0.021 ± 0.00 ^a^	0.033 ± 0.00 ^a^	0.024 ± 0.00 ^a^	-	<LOQ	<LOQ				9
**Dose**	**Pesticide**	**PHI (Days) (mg·kg^−1^)**						**MRL (mg·kg^−1^)**			**Recommended PHI (Days)**
		**2 h**	**1**	**3**	**5**	**7**	**14**	**China**	**CAC**	**EU**	
Recommended dose	Chlorantraniliprole (SC)	2.70 ± 0.08 ^b^	2.64 ± 0.11 ^b^	2.28 ± 0.09 ^b^	2.58 ± 0.21 ^b^	2.43 ± 0.07 ^b^	1.78 ± 0.00 ^a^	7 *	7	0.06	31
1.5-fold recommended dose		4.96 ± 0.26 ^a^	3.99 ± 0.17 ^a^	3.70 ± 0.25 ^a^	3.48 ± 0.07 ^a^	2.80 ± 0.04 ^a^	1.85 ± 0.08 ^a^				(7) 31

PHI: the pre-harvest interval. “*” represents a temporary limit value. GR = granules, SC = Suspension concentrate. Different letters across treatments indicate significant differences at *p* < 0.05.

**Table 5:** Residue levels at different pre-harvest intervals after applying the recommended dose combined with 2 g·ha^−1^ Se nanoparticles.

Treatment	Pesticide	PHI (Days) (mg·kg^−1^)						MRL (mg·kg^−1^)			Recommended PHI (Days)
		2 h	1	3	5	7	14	China	CAC	EU	
Control	Thiamethoxam (SC)	0.101 ± 0.01 ^b^	0.084 ± 0.01 ^a^	0.044 ± 0.01 ^a^	0.020 ± 0.00 ^a^	0.011 ± 0.00	<LOQ	1	1	0.01	7
Se nanoparticles		0.131 ± 0.01 ^a^	0.073 ± 0.01 ^a^	0.037 ± 0.00 ^a^	0.017 ± 0.00 ^a^	<LOQ	<LOQ				7
Control	Abamectin (EC)	0.219 ± 0.04 ^b^	0.139 ± 0.02 ^a^	0.025 ± 0.00 ^a^	<LOQ	<LOQ	<LOQ	0.05	0.03	0.01	2
Se nanoparticles		0.351 ± 0.03 ^a^	0.166 ± 0.02 ^a^	0.014 ± 0.00 ^a^	0.011 ± 0.00	<LOQ	<LOQ				3
Control	Imidacloprid (SC)	4.446 ± 0.30 ^a^	2.565 ± 0.26 ^a^	1.263 ± 0.08 ^a^	0.338 ± 0.03 ^a^	0.301 ± 0.03 ^a^	0.034 ± 0.01 ^a^	5	6	0.5	2
Se nanoparticles		4.613 ± 0.07 ^a^	2.154 ± 0.11 ^b^	0.803 ± 0.06 ^b^	0.529 ± 0.01 ^a^	0.281 ± 0.01 ^a^	0.030 ± 0.01 ^a^				2
Control	Lambda-cyhalothrin (SC)	0.687 ± 0.03 ^a^	0.585 ± 0.01 ^a^	0.601 ± 0.02 ^a^	0.151 ± 0.03 ^a^	0.175 ± 0.03 ^a^	0.118 ± 0.03 ^a^	0.5	/	0.07	2
Se nanoparticles		0.709 ± 0.06 ^a^	0.621 ± 0.07 ^a^	0.578 ± 0.02 ^a^	0.196 ± 0.02 ^a^	0.191 ± 0.05 ^a^	0.035 ± 0.02 ^b^				3
Control	Acetamiprid (WDG)	0.042 ± 0.00 ^a^	0.031 ± 0.01 ^b^	0.013 ± 0.00 ^b^	0.016 ± 0.00 ^a^	0.011 ± 0.00 ^b^	<LOQ	3	1.5	0.01	1
Se nanoparticles		0.040 ± 0.00 ^a^	0.039 ± 0.01 ^a^	0.023 ± 0.01 ^a^	0.017 ± 0.00 ^a^	0.024 ± 0.00 ^a^	<LOQ				1

Note: EC = Emulsifiable Concentrate, SC = Suspension concentrate, WDG = Water dispersible granules. Different letters across treatments indicate significant differences at *p* < 0.05.

**Table 6:** Residual kinetics and half-life of four pesticides in celery under recommended dose and 1.5-fold recommended dose conditions.

Pesticide	Sample Type	Dosage	Initial Residue (mg·kg^−1^)	Final Residue (mg·kg^−1^)	Dissipation Kinetics	Correlation Coefficient (R)	Half-Life (Day)
Clothianidin (GR)	Celery	Recommended dose	0.060 ± 0.00 ^b^	<LOQ	Ct = 0.059 × 10^−0.04t^	0.78	16.9
		1.5-fold recommended dose	0.108 ± 0.00 ^a^	0.013 ± 0.00	Ct = 0.119 × 10^−0.05t^	0.81	15.8
	Soil	Recommended dose	0.705 ± 0.10 ^b^	0.015 ± 0.00 ^a^	Ct = 3.632 × 10^−0.11t^	0.96	21.2
		1.5-fold recommended dose	1.238 ± 0.22 ^a^	0.012 ± 0.00 ^a^	Ct = 5.636 × 10^−0.13t^	0.86	17
Imidacloprid (GR)	Celery	Recommended dose	<LOQ	0.404 ± 0.01 ^a^	Ct = 8.619 × 10^−0.04t^	0.99	46.5
		1.5-fold recommended dose	0.029 ± 0.00	0.246 ± 0.06 ^b^	Ct = 22.670 × 10^−0.05t^	0.91	45.5
	Soil	Recommended dose	0.083 ± 0.01 ^a^	0.423 ± 0.03 ^b^	Ct = 23.988 × 10^−0.07t^	0.99	37.9
		1.5-fold recommended dose	0.154 ± 0.05 ^a^	1.991 ± 0.11 ^a^	Ct = 75.784 × 10^−0.07t^	0.98	40.3
Abamectin (GR)	Celery	Recommended dose	0.021 ± 0.00 ^a^	<LOQ	Ct = 0.035 × 10^−0.04t^	0.99	33.1
		1.5-fold recommended dose	0.021 ± 0.00 ^a^	<LOQ	Ct = 0.041 × 10^−0.02t^	0.97	39.8
	Soil	Recommended dose	0.141 ± 0.01 ^b^	0.163 ± 0.01 ^b^	Ct = 1.907 × 10^−0.03t^	0.94	55.9
		1.5-fold recommended dose	0.279 ± 0.02^a^	1.389 ± 0.08 ^a^	Ct= 3.611 × 10^−0.02t^	0.99	70.3
Chlorantraniliprole (SC)	Celery	Recommended dose	2.701 ± 0.08^b^	1.784 ± 0.00 ^a^	Ct= 2.766 × 10^−0.03t^	0.98	-
		1.5-fold recommended dose	4.958 ± 0.26^a^	1.847 ± 0.08 ^a^	Ct= 4.614 × 10^−0.07t^	0.97	-

Note: GR = granules, SC = Suspension concentrate. Different letters across treatments indicate significant differences at *p* < 0.05.

**Table 7:** Dissipation kinetics and half-lives of five pesticides in celery at the recommended dose combined with 2 g·ha^−1^ Se nanoparticles.

Pesticide	Treatment	Initial Residue (mg·kg^−1^)	Final Residue (mg·kg^−1^)	Dissipation Kinetics	Correlation Coefficient (R)	Half-Life (Day)
Thiamethoxam (SC)	Control	0.101 ± 0.01 ^b^	<LOQ	Ct = 0.109 × 10^−0.32t^	0.99	2.4
	Se nanoparticles	0.131 ± 0.01 ^a^	<LOQ	Ct = 0.103 × 10^−0.33t^	0.99	1.4
Abamectin (EC)	Control	0.219 ± 0.04 ^b^	<LOQ	Ct = 0.093 × 10^−0.34t^	0.86	0.5
	Se nanoparticles	0.351 ± 0.03 ^a^	<LOQ	Ct = 0.198 × 10^−0.52t^	0.98	0.2
Imidacloprid (SC)	Control	4.446 ± 0.30 ^a^	0.034 ± 0.01 ^a^	Ct = 3.365 × 10^−0.34t^	0.98	1.2
	Se nanoparticles	4.613 ± 0.07 ^a^	0.030 ± 0.01 ^a^	Ct = 3.158 × 10^−0.34t^	0.99	0.9
Lambda-cyhalothrin (SC)	Control	0.687 ± 0.03 ^a^	0.118 ± 0.03 ^a^	Ct = 0.595 × 10^−0.13t^	0.86	4.2
	Se nanoparticles	0.709 ± 0.06 ^a^	0.035 ± 0.02 ^b^	Ct = 0.797 × 10^−0.22t^	0.98	3.7
Acetamiprid (WDG)	Control	0.042 ± 0.00 ^a^	<LOQ	Ct = 0.036 × 10^−0.19t^	0.97	2.9
	Se nanoparticles	0.040 ± 0.00 ^a^	<LOQ	Ct = 0.042 × 10^−0.14t^	0.95	5.3

Note: SC = Suspension concentrate, EC = Emulsifiable Concentrate, WDG = Water dispersible granules. Different letters across treatments indicate significant differences at *p* < 0.05.

**Table 8:** Risk quotient and risk probability of 4 pesticides in celery after application at the recommended dose and 1.5-fold the recommended dose.

Pesticide	Treatment	STMR (mg·kg^−1^)	ADI (mg·kg^−1^ bw)	ARfD (mg·kg^−1^ bw)	RQ c%	RQ a%
Clothianidin (GR)	Recommended dose	0.010	0.1	0.6	9.85	1.64
	1.5-fold recommended dose	0.021			9.86	1.64
Imidacloprid (GR)	Recommended dose	0.40	0.06	0.4	22.83	3.34
	1.5-fold recommended dose	0.23			22.42	3.42
Abamectin (GR)	Recommended dose	0.016	0.001	-	116.47	-
	1.5-fold recommended dose	0.024			117.63	-
Chlorantraniliprole (SC)	Recommended dose	2.46	2	-	3.83	-
	1.5-fold recommended dose	3.52			3.90	-

STMR: supervised trials median residue; ADI: acceptable daily intake; ARfD: acute reference dose; RQ c: the chronic dietary exposure risk probability; RQ a: the acute dietary exposure risk probability.

**Table 9:** Risk quotient and risk probability of 5 pesticides in celery after pesticide application at the recommended dose combined with 2 g·ha^−1^ Se nanoparticles.

Pesticide	Treatment	STMR (mg·kg^−1^)	ADI (mg·kg^−1^ bw)	ARfD (mg·kg^−1^ bw)	RQ c%	RQ a%
Thiamethoxam (SC)	Control	0.046	0.08	1.0	7.61	0.61
	Se nanoparticles	0.050			7.62	0.61
Abamectin (EC)	Control	0.13	0.001	-	133.03	-
	Se nanoparticles	0.08			125.77	-
Imidacloprid (SC)	Control	0.78	0.06	0.4	23.75	3.56
	Se nanoparticles	0.64			23.41	3.51
Lambda-cyhalothrin (SC)	Control	0.18	0.02	0.02	63.30	63.30
	Se nanoparticles	0.40			64.90	64.90
Acetamiprid (WDG)	Control	0.016	0.07	0.1	32.48	22.74
	Se nanoparticles	0.028			32.51	22.76

## Data Availability

The data presented in this study are available on request from the corresponding author.

## Supplementary Materials

The following are available online at https://www.mdpi.com/article/10.3390/foods10091987/s1, Table S1: Pesticide labeling information and recommended dose, Table S2: Pesticide labeling information and recommended dose combined with 2 g·ha^−1^ Se nanoparticles, Table S3: Instrument parameters and their physicochemical properties.
