# Supplementary material for: Pesticide Residue Behavior and Risk Assessment in Celery after Se Nanoparticles Application
Source: Foods. 2021 Aug 25;10(9):1987. doi: 10.3390/foods10091987 (PMC8470415; doi:10.3390/foods10091987)
Supplement: Supplementary file 1 [file foods-10-01987-s001.zip › foods-1318323-supplementary.pdf]

Supplementary Materials

Article

Pesticide Residue Behavior and Risk Assessment in Celery after Se Nanoparticles Application

Lu Kang [1,2], Hejiang Liu [2], Duoyong Zhao [2], Canping Pan [1,*] and Cheng Wang [2,*]

[1] Innovation Center of Pesticide Research, Department of Applied Chemistry, College of Science, China
Agricultural University, Beijing 100193, China; 96208zx@163.com

[2] Key Laboratory of Agro-Products Quality and Safety of Xinjiang, Institute of Agricultural Quality Standards and Testing Technology, Xinjiang Academy of Agricultural Sciences, Urumqi 830091, China; liuhejiang2025@163.com (H.L.); luckydyz@163.com (D.Z.)

* Correspondence: canpingp@cau.edu.cn (C.P.); wangcheng312@sina.com (C.W.); Tel.: +86-10-6273-1978 (C.P.); +86-991-4502-047 (C.W.); Fax: +86-10-62733620 (C.P.)

Table S1. Pesticide labeling information and recommended dose.

Serial Number	Pesticide Labeling Information	Pesticide Active Ingredient	Pesticide Type	Pesticide Application Dose	
				Recommended Dose	1.5-fold Recommend Dose
1	abamectin GR	abamectin (0.5%)	insecticide	16.9 g a.i./ha	19.7 g a.i./ha
2	imidacloprid GR	imidacloprid (5%)	insecticide	56.2 g a.i./ha	84.4 g a.i./ha
3	clothianidin GR	clothianidin (0.5 %)	insecticide	16.8 g a.i./ha	23.6 g a.i./ha
4	chlorantraniliprole SC	chlorantraniliprole (5%)	insecticide	6.8 g a.i./ha	10.1 g a.i./ha

Note: Active ingredient per ha (g a.i./ha). GR= granules.

Table S2. Pesticide labeling information and recommended dose combined with 2 g·ha^{-1} Se nanoparticles.

Serial Bumber	Pesticide Labeling Information	Pesticide Active Ingredient	Pesticide Type	Pesticide Recommended Dose	
				Control	Se Nanoparticle
1	abamectin EC	abamectin (3.2 %)	insecticide	1.6 g a.i./ha	1.6 g a.i./ha
2	imidacloprid SC	imidacloprid (20%)	insecticide	6.8 g a.i./ha	6.8 g a.i./ha
3	acetamiprid WDG	acetamiprid (70%)	insecticide	2.0 g a.i./ha	2.0 g a.i./ha
4	thiamethoxam SC<br>lambda-cyhalothrin SC	thiamethoxam (12.6%)<br>lambda-cyhalothrin (9.4%)	insecticide<br>insecticide	1.0 g a.i./ha<br>0.7 g a.i./ha	1.0 g a.i./ha<br>0.7 g a.i./ha

Note: EC= Emulsifiable Concentrates, SC = Suspension concentrate, WDG= Water dispersible granules.

Table S3. Instrument parameters and their physicochemical properties.

Pesticide Active Ingredient		Retention Time (min)	Quantitative Ion (m/z)	Qualitative Ion (m/z)	Collision Energy (eV)	Molecular Weight	Log Kow	Melting Point (°C)	Vapor Pressure (mPa, 20 °C)
A	Clothianidin	3.09	250/93.1 *	250/132	13;45	249.7	0.7	176.8	3.8×10^{-8}
	Imidacloprid	8.42	256.1/174.9 *	256.1/209.1	20;20	255.7	0.57	144.0	4×10^{-7}
	Thiamethoxam	9.32	306.2/200.9 *	306.2/115.6	12;16	291.7	−0.13	139.1	6.6×10^{-6}
	Acetamiprid	10.50	223.0/125.6 *	223.0/89.6	36;23	222.7	0.80	98.9	1×10^{-3}
	Chlorantraniliprole	11.79	484.0/285.9 *	484.0/453.0	22;22	483.2	2.8	208-210	6.3×10^{-9}
	Abamectin	14.77	895.6/751.7 *	895.6/327.4	41;51	873.1	4.4	162-169	3.7×10^{-3}
B	Lambda-cyhalothrin	31.6;31.9	197.0/141.0 *	197.0/161.0	10;5	449.9	7.0	49.2	2×10^{-4}

A: Pesticides analyzed by LC-MS/MS; B: Pesticide analyzed by GC-MS/MS. "*" represent quantitative ion.
